# *In Vitro* Induced Regulatory T Cells Are Unique from Endogenous Regulatory T Cells and Effective at Suppressing Late Stages of Ongoing Autoimmunity

**DOI:** 10.1371/journal.pone.0104698

**Published:** 2014-08-13

**Authors:** Thanh-Long M. Nguyen, Nabil T. Makhlouf, Bryan A. Anthony, Ryan M. Teague, Richard J. DiPaolo

**Affiliations:** Department of Molecular Microbiology and Immunology, Saint Louis University School of Medicine, Saint Louis, Missouri, United States of America; New York University, United States of America

## Abstract

Strategies to boost the numbers and functions of regulatory T cells (Tregs) are currently being tested as means to treat autoimmunity. While Tregs have been shown to be effective in this role, strategies to manipulate Tregs to effectively suppress later stages of ongoing diseases need to be established. In this study, we evaluated the ability of TGF-β-induced Tregs (iTregs) specific for the major self-antigen in autoimmune gastritis to suppress established autoimmune gastritis in mice. When transferred into mice during later stages of disease, iTregs demethylated the Foxp3 promoter, maintained Foxp3 expression, and suppressed effector T cell proliferation. More importantly, these iTregs were effective at stopping disease progression. Untreated mice had high numbers of endogenous Tregs (enTregs) but these were unable to stop disease progression. In contrast, iTregs, were found in relatively low numbers in treated mice, yet were effective at stopping disease progression, suggesting qualitative differences in suppressor functions. We identified several inhibitory receptors (LAG-3, PD-1, GARP, and TNFR2), cytokines (TGF-β1 and IL12p35), and transcription factors (IRF4 and Tbet) expressed at higher levels by iTregs compared to enTregs isolated form mice with ongoing disease, which likely accounts for superior suppressor ability in this disease model. These data support efforts to use iTregs in therapies to treat establish autoimmunity, and show that iTregs are more effective than enTregs at suppressing inflammation in this disease model.

## Introduction

Regulatory T cells (Tregs) are important for regulating immune responses and maintaining immunological homeostasis. The transcription factor, forkhead box P3 (Foxp3), is essential for the development, maintenance, and function of Tregs [1], [2]. Mice and humans that do not express functional Foxp3 protein develop systemic autoimmunity and die prematurely, emphasizing the importance of Tregs in maintaining immune homeostasis [3]–[6]. Decreased numbers and/or functions in Tregs is likely an important risk factor for developing autoimmunity, and strategies to improve Treg number and functions are currently being developed as a means to treat chronic inflammation, autoimmunity, and to prevent the rejection of transplanted tissue [7].

Tregs are categorized into different populations depending on their origin and on the method by which Foxp3 expression is induced [8]. *In vivo*, Tregs are generated in the thymus (tTregs) and can be induced in the peripheral tissues (pTregs). Both tTregs and pTregs are important for maintaining immune tolerance [9]. Tregs can also be induced *in vitro* by stimulating naïve T cells in the presence of TGF-β1 and IL-2 (iTregs) [10], [11]. The adoptive transfer of iTregs is an attractive therapeutic strategy because iTregs can be induced and expanded to large numbers prior to transfer. While iTregs have been shown to be an effective treatment in the early stages of mouse models of human diseases [12]–[16], there is controversy over whether iTregs retain Foxp3 expression and suppressor functions after transfer into various environments [7]. Some reports have shown that iTregs retain Foxp3 expression and are resistant to converting to effector T cells [17]–[19], while others report that under certain conditions iTregs lose Foxp3 expression and acquire effector functions [20], [21]. These differences may be explained, in part, by technical differences in how iTregs were generated, and in the disease setting in which they were transferred.

In this study, we evaluated whether antigen specific iTregs were effective at suppressing disease when transferred into mice during the late stages of autoimmune gastritis (AIG). TxA23 mice are T cell receptor (TCR) transgenic mice in which CD4^+^ T cells express a TCR specific for a peptide from the H^+^/K^+^ ATPase α chain expressed by parietal cells, which is the same auto-antigen targeted in the human disease [22], [23]. All mice spontaneously develop AIG by one month of age and ultimately develop gastric intraepithelial neoplasias (gastric cancer) by 12 months [24]. Importantly, we recently reported that disease progression in this model mimics many aspects of the development of gastric cancer in humans [25].

The goal of this study was to evaluate the ability of antigen specific iTregs to stop disease progression when transferred into mice during the advanced stages of AIG. We observed that after iTregs were transferred into mice with AIG, they demethylated the Treg specific demethylated region (TSDR) on the Foxp3 promoter, maintained Foxp3 protein expression, and were effective at suppressing inflammation and decreasing the severity of disease. Despite relatively low numbers of iTregs in treated TxA23 mice, they had superior suppressor functions in this environment compared to endogenous Tregs (enTregs), which were present at much higher numbers in the untreated mice. The superior suppressor function by iTregs correlated with higher expression of several surface receptors (LAG3, PD-1, GARP, and TNFR2), cytokines (TGF-β1, IL-12p35), and transcription factors (IRF4 and Tbet) compared to enTregs. The increased expression of these receptors, cytokines, transcriptions factors under these highly inflammatory conditions are likely to be responsible for the increased ability of iTregs to suppress AIG. Together, the data in this study demonstrates that iTregs were effective at suppressing disease progression during the late stages of autoimmune gastritis.

## Methods

### Mice

TxA23 TCR transgenic mice have been previously described [23]. BALB/c mice were purchased from Jackson Laboratories. TxA23 mice were bred to express Thy1.1/1.1, Thy1.1/1.2, and Foxp3.eGFP reporter [26]. All animals were maintained in the Department of Comparative Medicine, Saint Louis University School of Medicine in accordance with institutional guidelines. Saint Louis University School of Medicine Institutional Animal Care and Use Committee approved all procedures performed on mice (Protocol 1605). Mice were euthanized by CO_2_ asphyxiation followed by cervical dislocation. All efforts were made to minimize suffering.

### T cell purification and iTreg differentiation

iTregs were generated as previously described [11], [12]. Briefly, CD4 single-positive thymocytes were sorted from 6–8-wk-old TxA23 TCR transgenic mice and stimulated with plate-bound anti-CD3 (1 µg/ml) and anti-CD28 (2 µg/ml) (BD Pharmingen) in 24-well plates (2.5×10^5^ cells/well) in 2 ml complete RPMI media (modified RPMI 1640 supplemented by 10% FBS, 2 mM glutamine, 100 U/ml penicillin, 100 mg/ml streptomycin, 10 mM HEPES, 4×10^−7^ M 2-ME, 1 mM essential amino acids, and 1 mM sodium pyruvate; all from Sigma Aldrich, MO) supplemented with recombinant human IL-2 (100 U/ml). Recombinant human TGF-β1 (Miltenyi Biotech, Auburn, CA) (5 ng/ml) was added to induce iTregs. Cells were removed from plate-bound antibodies after 48 hours and added to new wells with fresh media supplemented with 100 U/ml recombinant human IL-2. Cells were used after 7 days in culture. The proportion of cells expressing Foxp3 after 7 days of culture was typically 60–80%.

### Histopathology

Stomachs were removed from mice, rinsed in saline, immersion fixed in 10% neutral-buffered formalin, paraffin embedded, sectioned, and stained with hematoxylin and eosin. Pathology scores were assigned using methods modified from Rogers and colleagues [27]. Slides were blinded, and sections from individual mice were assigned scores between 0 (absent) and 4 (severe) to indicate the severity of inflammation, parietal cell death, and mucosal hyperplasia.

### Isolation of cells from the gastric lymph nodes and gastric mucosa

The method for isolating cells from the stomach tissue has been described previously [28], [29]. Briefly, the gastric lymph nodes were removed from the stomachs, homogenized, and passed through a 40-µM-pore nylon filter. Stomachs were opened with an incision from the antrum to the fundus, and rinsed in PBS. Cells were flushed from the gastric mucosa using a syringe with a 25 gauge needle filled with cold PBS+5% FCS + penicillin/streptomycin to repeatedly inject fluid into the mucosa causing the tissue to swell and rupture. Single cell suspensions were collected, gently vortexed, and passed through a 40-µM nylon filter. Cells were counted, stained with antibodies, and analyzed by flow cytometry.

### Flow cytometry

Cell surface staining was performed according to standard procedures using antibodies against anti-mouse CD4 (clone GK1.5; BD Pharmingen), anti-mouse Thy1.1 (clone OX-7; BD Pharmingen), anti-mouse Thy1.2 (clone 53-2.1; BD Pharmingen), anti-mouse LAG-3 (clone C9B7W; BD Pharmingen), anti-mouse TNFR2 (clone TR75-89; BD Pharmingen), anti-mouse CD25 (clone PC61; BD Pharmingen), anti-mouse CTLA-4 (clone UC10-F10-11; BD Pharmingen), anti-mouse PD-1 (clone J43; eBioscience), anti-mouse GARP (clone YGIC86; eBioscience), anti-mouse CD38 (clone 90; eBioscience), anti-mouse GITR (clone DTA-1; eBioscience), anti-mouse neuropilin-1 (clone 3DS304M; eBioscience), and anti-mouse CD73 (cloneTY/11.8; Biolegend). Foxp3 was detected using the GFP reporter or by intracellular cytokine staining using a anti-mouse Foxp3 (clone FJK-16 s; eBioscience) antibody. All flow cytometry was performed on a BD LSRII or BD FACSCalibur and analyzed using FlowJo (TreeStar). Surface stain was performed by incubating cells with antibody cocktail mix for 20 minutes at 4°C. For intracellular cytokine Foxp3 staining, cells were first stained for surface receptors, fixed in 4% formyl saline, permeabilized (0.5% BSA, 0.1% Triton, and 2 mM EDTA in PBS) for 45 minutes at room temperature. Cells were incubated overnight with the anti-cytokine antibodies and analyzed by flow cytometry.

### Quantitative Real Time PCR

Total RNA was prepared using the RNeasy Mini Kit system (Qiagen). The quantity and quality of RNA was determined using a NanoDrop 2000 spectrophotometer (Thermo Scientific) cDNA copy of RNA isolated from cells was done according to the manufacturer's instruction (High Capacity cDNA Reverse Transcription Kit, Applied Biosystems). Quantitative PCR was performed using primer/probes purchased from Applied Biosystems. GAPDH served as an internal reference standard. PCR was run on the 7500 Real-Time PCR System (Applied Biosystems). The following primer/probe sets were used: *Tbx21* (Mm00450960_m1), *Irf4* (Mm00516431_m1), *Ebi3* (Mm00469294_m1), *Il12a* (Mm00434165_m1), *Tgfb1* (Mm01178820_m1), *Gata3* (Mm00484683_m1), *Il10* (Mm00439614_m1), *Blimp1* (Mm01187285_m1) and *Gapdh* (Mm99999915_g1).

### Foxp3 Stability Assays

iTregs were generated from TxA23-Thy1.1/1.2xFoxp3.eGFP mice. Seven days after culture, iTregs were sorted to 100% purity by sorting on CD4^+^Foxp3.eGFP^+^ cells. enTregs were sorted from the gastric lymph node of TxA23-Thy1.1/1.2 Foxp3.eGFP mice to 100% purity by sorting on CD4^+^Foxp3.eGFP^+^ cells. The sorted iTregs and enTregs were then injected into TxA23 Thy1.1/1.1xFoxp3.eGFP mice. Cells were isolated from the stomach 1 week after transfer. The percentages of Thy1.2^+^Foxp3.eGFP^+^ cells were analyzed by Flow Cytometry. Methylation at the TSDR was evaluated by quantitative PCR as described in Yadev et. al.[30].

### *In Vivo* Suppression Assay

iTregs were generated from TxA23-Thy1.1/1.1 mice. 5×10^6^ cells were injected I.P. into 3 TxA23-Thy1.1/1.1 mice and 3 TxA23-Thy1.1/1.1 mice were injected with PBS. Two days after iTreg injection, 1×10^6^ CFSE labeled effector T cells were injected I.V. into all mice. Effector T cells were isolated from the gastric lymph node and spleen of TxA23-Thy1.1/1.2 mice using Miltenyi AutoMACS beads. Gastric lymph node and spleen were processed into a single cell suspension. Total T cells were isolated using a PAN-TII isolation kit (Miltenyi). Tregs in the spleen and gastric lymph node were removed by labeling cells with an anti-CD25-PE and then using anti-PE beads (Miltenyi). The remaining CD4 cells were then labeled with Carboxyfluorescein succinimidyl ester (CFSE) proliferation dye prior to injection. One week after injection of effector T cells, the gastric lymph node was isolated from both iTreg treated and PBS treated groups. Proliferation of transferred Thy1.2^+^ effector T cells was determined by dilution of CFSE dye using Flow Cytometry.

### Statistical Analysis

Data are expressed as means of individual determinations +/− standard error. Statistical analysis was performed using the Mann-Whitney Test (*P<.05; **P<.01; ***P<.001) using GraphPad Prism 5.

## Results

### iTregs demethylate the Foxp3 promoter and retain Foxp3 expression after transfer

**Figure 1:**
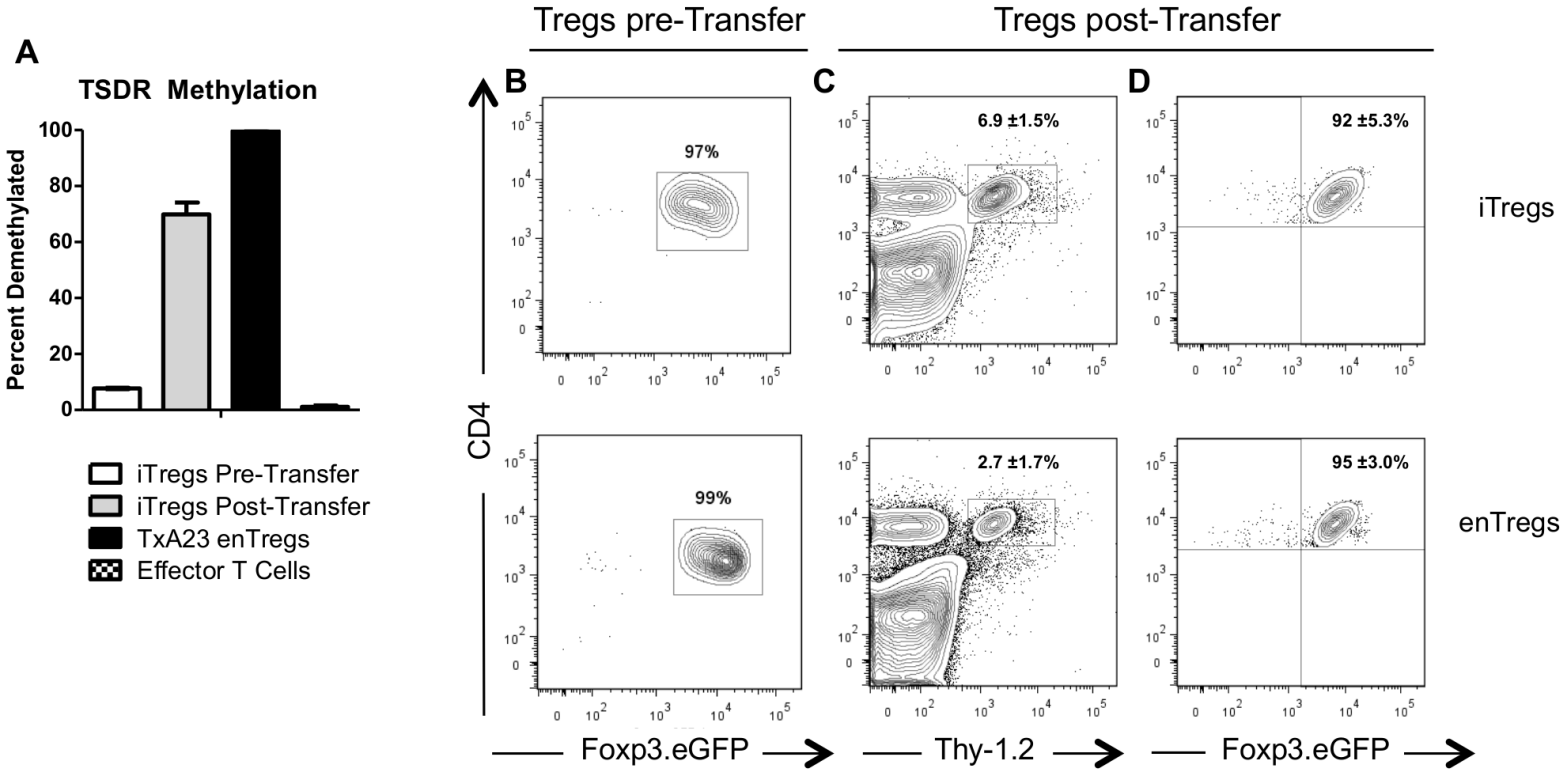
iTregs demethylated the Treg specific demethylated (TSDR) region and maintained Foxp3 expression. (A) The methylation status of the Foxp3 TSDR of iTregs prior to and after transferring into TxA23 mice. Also shown are enTregs (CD4^+^Foxp3^+^) and conventional (CD4^+^Foxp3^-^) cells from TxA23 mice. Flow cytometric analysis of (B) the purity of enTregs and iTregs prior to transferring into TxA23 mice, (C) the percentage of Thy1.2^+^ transferred Tregs in the gastric mucosa, and (D) the expression of Foxp3.eGFP by the Thy1.2^+^ Tregs one week after transfer. Data shown represents 2 mice for the enTreg group and 3 mice for the iTreg group from two independent experiments.

An important consideration for the use of Tregs in cell-based therapies is the stability of Foxp3 expression, which is essential for Treg suppressor functions [2]. The degree of methylation at the Treg specific demethylated region (TSDR) of the Foxp3 promoter influences the stability of Foxp3 expression [20]. This is particularly true for TGF-β induced Tregs, where methylation of the TSDR has been associated with a loss Foxp3 expression [31]. Therefore, we determined the methylation status of the Foxp3 TSDR in of H^+^/K^+^ ATPase iTregs before and 1 week after transfer into TxA23 mice with ongoing autoimmune gastritis. Transferred iTreg were tracked via a Thy-1.2 congenic marker and expression of a Foxp3-IRES-eGFP reporter. Consistent with previously published results [20], [31], the TSDR of iTregs was highly methylated after *in vitro* induction, similar to Foxp3-negative T cells (Figure 1A). However, after transfer into TxA23 mice, re-isolated iTreg showed approximately 75% demethylation of the TSDR (Figure 1A). For comparison, the TSDR of endogenous Tregs (enTregs) from untreated TxA23 mice was completely demethylated. These data showed that although iTregs entered TxA23 mice with a completely methylated TSDR region, this region was demethylated after they were transferred.

To determine if iTregs maintained Foxp3 expression in the highly inflammatory environment associated with ongoing autoimmune gastritis, iTregs were induced from TxA23xFoxp3-IRES-eGFP reporter mice and enTregs were isolated from the gastric lymph node TxA23xFoxp3-IRES-eGFP reporter mice, both on a Thy1.2 congenic background. Cells were sorted to >97% purity by selecting for GFP+ cells (Figure 1B), and adoptively transferred into TxA23 mice. One week after transfer, cells were isolated from the gastric mucosa of TxA23 recipient mice and the expression of Foxp3 expression was assessed in the transferred cells (Figure 1C). Approximately 92% of transferred iTregs located in the stomach expressed Foxp3.eGFP (Figure 1D), indicating that iTregs retained Foxp3 expression after transfer into mice with established AIG. Similarly, approximately 95% of transferred enTregs located in the stomach expressed Foxp3.eGFP. Together these data show that iTregs demethylated the Foxp3 TSDR and maintained Foxp3 expression when transferred into mice with ongoing AIG.

### iTregs suppress effector T cell proliferation and stop disease progression

**Figure 2:**
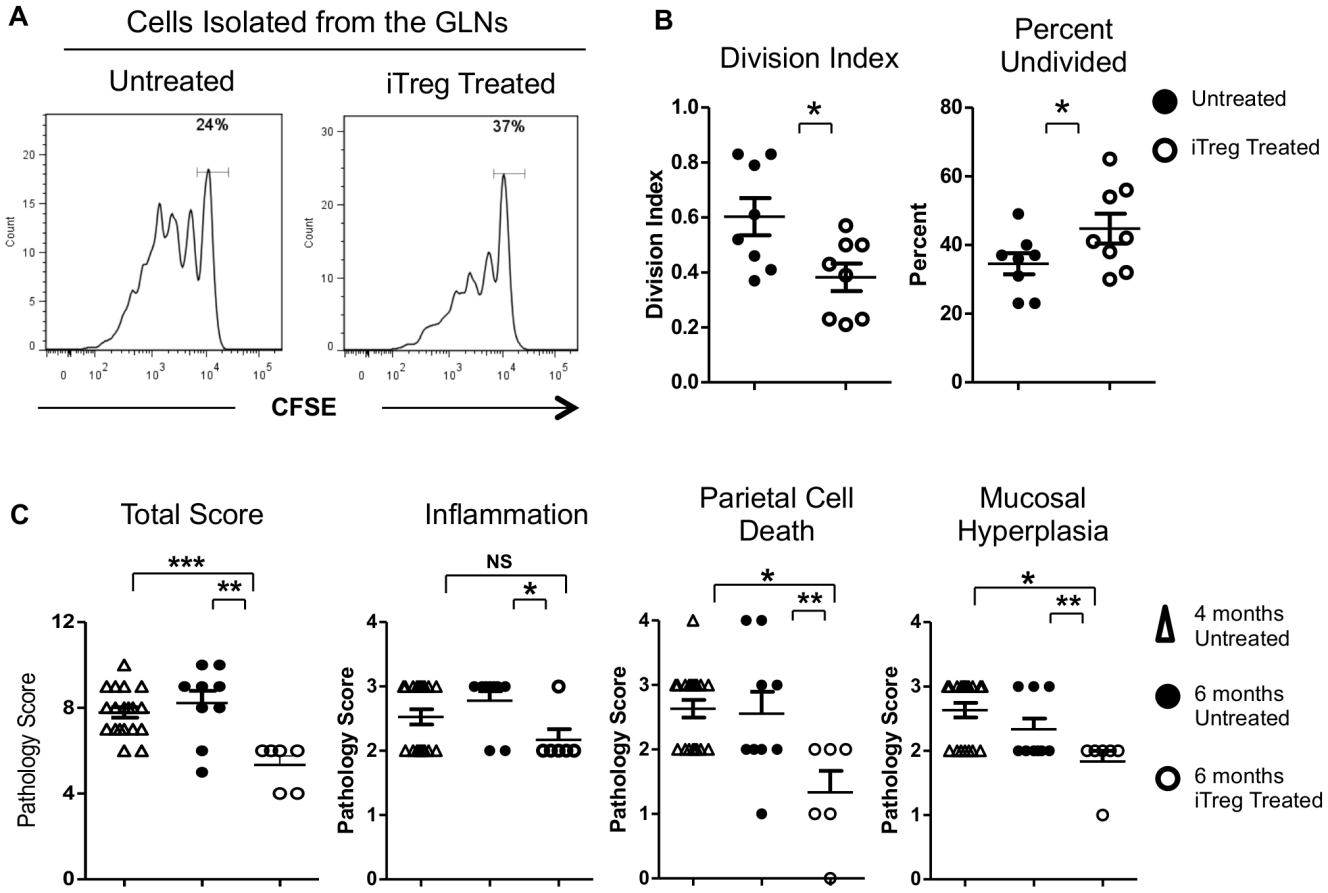
Treatment with iTregs suppressed effector T cell proliferation and reversed disease pathology in TxA23 mice. (A) Representative histograms of the CFSE dilution by labeled T cells transferred into untreated and iTreg mice. Data represents the mean ± SEM of a combination of two independent experiments with 4 mice in each group. (B) Division index and percent undivided of CFSE labeled effector T cells from individual mice. (C) Pathology scores of stomachs from 4-month-old TxA23 mice, 6-month-old TxA23 mice that were left untreated, and 6-month-old mice that received 5×10^6^ H^+^/K^+^ ATPase-specific iTregs at 4 months of age. Sections from individual mice were graded on a scale of 0–4 for severity of inflammation, parietal cell death, and mucosal hyperplasia. The total pathology score is the sum of the individual scores for each individual mouse. Data for 6-month-old untreated and iTreg treated mice represents the mean ± SEM of a combination of two independent experiments with three mice per group. Data were analyzed using a Mann–Whitney *U* test *p<0.05, **p<.01, ***p<.001.

iTregs are effective at suppressing inflammation and preventing the progression of different mouse model of autoimmune disease when administered at early stages of disease [7]. However, the effectiveness of iTregs to suppress inflammation during later stages of disease is not well established. To determine if iTregs were effective at suppressing inflammation at late stage of AIG, 4-month-old TxA23 mice were treated with 2.5×10^6^ H^+^/K^+^ ATPase specific iTregs. At this age, TxA23 mice have severe inflammation, hyperplasia, and significant loss of parietal cells in the gastric mucosa [24]. To assess their ability to suppress effector T cell proliferation, 1×10^6^ H^+^/K^+^ ATPase-specific CD4^+^Foxp3^-^ effector T cells expressing a Thy-1.2 congenic marker and labeled with CFSE proliferation tracking dye were transferred into iTreg-treated TxA23 mice and untreated TxA23 mice. After 7 days, cells were isolated from the gastric lymph nodes and proliferation of effector T cells was determined by CFSE dilution. There was a significant reduction in both the proportion of cells that divided, and in the average number of divisions by effector T cells transferred into iTreg pre-treated mice compared to untreated TxA23 mice (Figure 2A and B). These data indicate that transferred iTregs were effective at suppressing effector T cell proliferation in mice with ongoing AIG.

Having shown that iTregs were effective at suppressing effector T cells proliferation in 4-month-old TxA23 mice, we next determined whether iTreg-treatment was effective at decreasing the severity of gastric pathology. 5×10^6^ iTregs were adoptively transferred into 4-month-old TxA23 mice. After 2 months, when the mice were 6 months of age, stomachs were isolated and the severity of disease was compared between iTreg-treated and age matched untreated TxA23 mice. Mice treated with iTregs had significantly less inflammation, parietal cell death, and mucosal hyperplasia (Figure 2C). In fact, disease severity of 6-month-old mice treated with iTregs was significantly lower than it was at the time of treatment (4 months of age). These data indicate that iTregs were not only effective at stopping the progression of autoimmune gastritis, but reversed gastric pathology in mice with established AIG.

### Expression of surface markers, cytokines, and transcription factors associated with Treg suppressor functions

**Figure 3:**
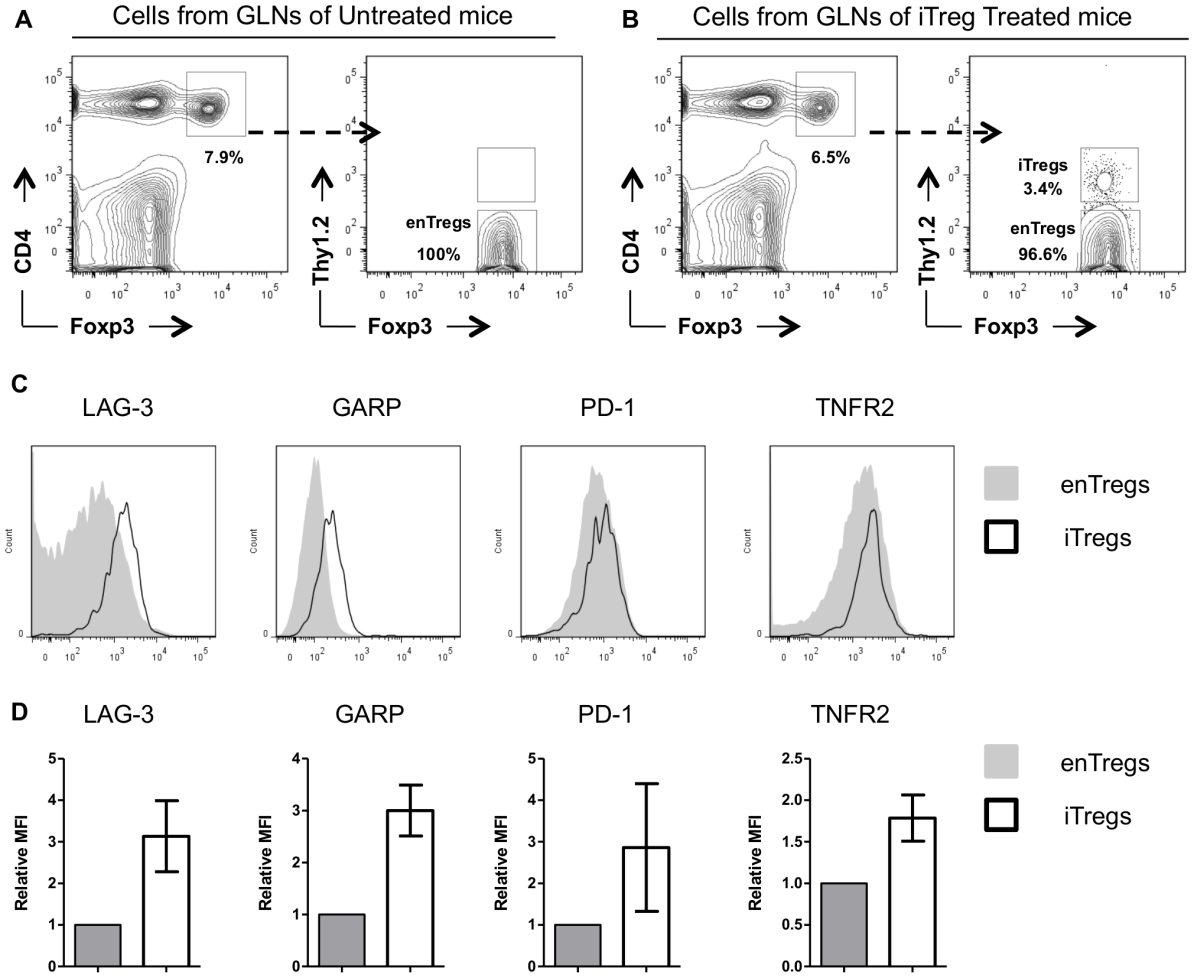
Comparison of inhibitory receptors expressed by enTregs and iTregs. (A–B) Representative flow cytometry plots measuring the percentage Foxp3^+^ enTregs and adoptively transferred iTregs (Foxp3^+^Thy1.2^+^) in the gastric lymph node of untreated and iTreg-treated TxA23 mice. (C) Representative histograms generated using flow cytometric analysis of surface receptors expressed by iTregs (black line) isolated from the gastric lymph nodes TxA23 mice 1 week after treated and enTregs (gray filled line) from age matched untreated TxA23 mice. (D) The mean fluorescent intensity (MFI) of individual receptors expressed by iTregs normalized to the expression by enTregs in individual experiments. Data represents an aggregation of 2–4 individual experiments.

During these analyses we observed that there were large numbers of enTregs present in untreated TxA23 mice (Figure 3A). However, these enTregs were less effective than iTregs at suppressing effector T cell proliferation and did not stop the progression of AIG (Figure 2A). In iTreg-treated TxA23 mice, the iTregs represented only a small percentage (∼3.4%) of all Tregs found in the gastric lymph node and stomach (Figure 3B), yet they were able to decrease effector T cell proliferation and suppressed disease pathology. These data suggested qualitative differences that endowed iTregs with superior suppressor functions compared to enTregs in this inflammatory setting.

Tregs use multiple mechanisms to mediate suppression, including expression of inhibitory receptors on their cell surface [32]. To determine whether iTregs expressed higher levels of inhibitory receptors, we compared expression levels of a panel of inhibitory receptors between enTregs from untreated TxA23 mice and iTregs that had been transferred into and then re-isolated TxA23 mice. Many of these receptors (CD73, CTLA4, neuropilin, CD25, GITR, and CD38)) were expressed at similar levels or at lower levels on iTregs compared to enTregs (Figure S1). However, iTregs expressed significantly higher levels of: lymphocyte-activation gene 3 (LAG-3), glycoprotein A repetitions predominant (GARP), programmed cell death protein 1 (PD-1), and TNF receptor 2 (TNFR2) [33]–[36] compared to enTregs (Figure 3C–D). To determine if the higher levels of expression of LAG-3, GARP, PD-1, and TNFR2 by iTregs was induced on iTregs during *in vitro* induction or after transfer into TxA23 mice, the expression levels of these receptors were compared prior to transfer and after iTregs were transferred and re-isolated from TxA23 mice. All four receptors were upregulated on iTregs after they were transferred into TxA23 mice (Figure S2). These data showed that iTregs expressed higher levels of many immunosuppressive receptors which were induced after transfer into the TxA23 mice.

**Figure 4:**
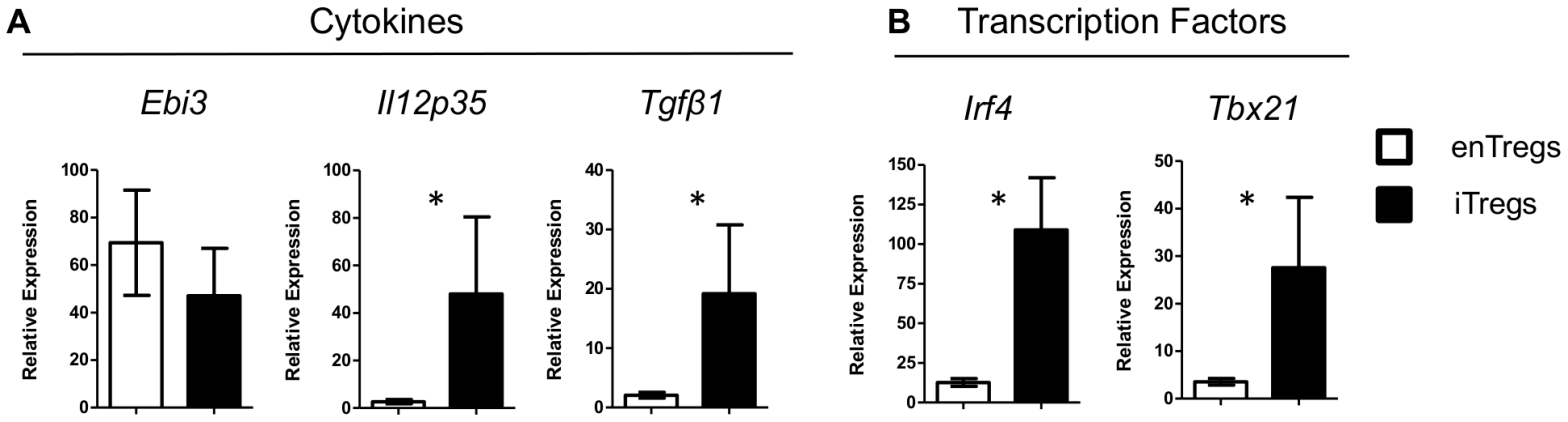
Comparison of cytokines and transcription factors expressed by enTregs and iTregs. mRNA was isolated from enTregs and iTregs that were transferred into TxA23 mice, analyzed by qRT-PCR and normalized to mRNA isolated from CD4^+^Foxp3^-^ T cells from BALB/c mice. (**A**) Comparison of expression levels of cytokines IL-35 (*Ebi3* and *Il12p35*) and *Tgfβ1*. (**B**) Comparison of expression levels of transcription factors *Irf4* and *Tbx21* (Tbet). Data shown as mean ± SEM from 4-6 independent experiments and analyzed using a Mann–Whitney *U* test. *p<0.05.

Tregs also mediate immunosuppression through the secretion of cytokines [37], [38]. Next we compared the relative expression levels of several immunosuppressive cytokines between enTregs from untreated TxA23 mice and iTregs that had been transferred into and re-isolated from TxA23 mice after 1 week. Quantitative real time PCR analysis (qRT-PCR) revealed a 10-fold increase expression of *Tgfβ1* and *Il12p35* in iTreg compared to enTregs (Figure 4A). *Ebi3* and *Il10* (Figure S3) were expressed at similar levels in iTregs and enTregs. It is possible that higher expression levels of these immunosuppressive cytokines, along with the increase expression of the inhibitory receptors, account for the increased suppressor functions of iTregs in TxA23 mice.

Finally, recent studies have shown that several transcription factors work in concert with Foxp3 to enable Tregs to effectively suppress different types of inflammation. For example, Tbet and IRF4 expression by Tregs influence their ability to suppress Th1 and Th2 mediated inflammation respectively [39], [40]. Lower expression of any of these transcription factors may result in a decrease in Treg suppressor functions. We compared the relative expression levels of transcriptions factors between enTregs and iTregs by qRT-PCR. iTregs expressed 10-fold higher levels both *Tbx21* (Tbet) and *Irf4* (Figure 4B). Many other transcription factors were expressed at similar levels (*Blimp1* and *Gata3*, Figure S3).

## Discussion

In these studies, we demonstrated that the adoptive transfer of antigen specific TGFβ induced Tregs were effective at stopping the progression of disease in TxA23 mice at the later stages of AIG (Figure 2). Although iTregs have been demonstrated to be effective at preventing various diseases in mouse models of human disease when given prior to or very shortly after disease induction [10], [14], [15], [41]–[43], few have examined the ability of iTregs to suppress autoimmunity at the late stage of disease. At the time of treatment, 4-months of age, TxA23 have developed pathological changes and molecular biomarkers seen in humans with chronic gastric inflammation. This is important as our experiments tested the effectiveness of iTregs to suppress disease during later stages of established autoimmunity. We demonstrated that not only were iTregs effective at suppressing disease, they had therapeutic effects as pathology scores of iTreg treated mice were lower than at the time of treatment. iTregs did not appear to alter the activity of enTregs in treated mice, as receptor expression profiles, frequencies, and total numbers of enTregs were similar in untreated and iTreg-treated mice. The observation that iTregs represented a very small proportion of all CD4 T cells in TxA23 mice where the majority of CD4 T cells were autoreactive highlights the potent suppressive capabilities of iTregs.

We showed that a large majority of iTregs demethylated the Foxp3 TSDR after transfer, and retained Foxp3 expression and suppressor functions (Figure 1). Concerns have been raised over whether or not various types of Tregs retain Foxp3 expression and suppressor activities in different inflammatory conditions. There are reports that Tregs lose their suppressor capabilities, and in some cases, convert into effector cells, referred to as ‘ex-Tregs’, that contribute to disease [44]–[49]. This has been a concern, specifically with iTregs, as they have been shown to lose Foxp3 expression under non-inflammatory conditions [20], [31], [50]. The results presented in this study are in agreement with other studies that showed that iTregs maintained Foxp3 expression under inflammatory conditions [12], [21].

We showed that treatment with iTregs suppressed autoreactive T cell proliferation in TxA23 mice. It is possible that iTregs were effective at suppressing T cell expansion due to their increased expression of inhibitory receptors LAG-3, GARP, and PD1 as compared to enTregs in untreated mice (Figure 3C). LAG-3 binds to MHC-class II molecules on immature and suppresses DC maturation by reducing CD80 and CD86 expression [35]. In fact, we previously reported that the same iTregs used in these studies inhibit the expression of co-stimulatory molecules CD80 and CD86 on dendritic cells [12]. In addition to the inhibitory receptors, iTregs also expressed higher levels TGF-β1 (Figure 4). GARP regulates the amount of surface bound TGF-β1 present on a cell [33]. The higher expression TGF-β1 in addition to the higher expression of GARP could allow for more latent TGF-β1 to be bound on the surface Tregs which could augment iTreg use of TGF-β1 to directly inhibit dendritic cell and T cells function [51].

Finally, we showed that iTregs expressed significantly higher levels of *Irf4* and *Tbet* compared to enTregs isolated from the same inflammatory environment (Figure 4). Foxp3 works in concert with a network of transcription factors to induced full suppressive functions in Tregs [39], [40], [52]–[54]. The increased expression of *Tbet* may enhance iTregs to suppress the Th1 mediated inflammation in TxA23 by increasing their expression of Tbet target genes including CXCR3, CCL3, and CCL4, allowing iTregs to migrate to the site of inflammation. We previously reported that the same iTregs secreted CCL3 and CCL4 and recruited additional iTregs into the gastric mucosa in TxA23 mice [55].

In summary, this study demonstrated that the adoptive transfer of iTregs late in disease progression of AIG was effective at suppressing established inflammation and reversing gastric pathology. iTregs acquired a different molecular phenotype, including demethylating the Foxp3 TSDR and up-regulating receptors associated with Treg suppressor functions, after they were transferred into the inflammatory environment of TxA23 mice. The observation that iTregs were still present in the gastric lymph node and stomach, expressed Foxp3, and represented a small fraction of total CD4 T cells two months after treatment provides evidence that iTregs have long-term therapeutic effects in this disease model. Overall, we have demonstrated that antigen-specific iTregs represented a highly suppressive and stable cell population supporting their use as a potential therapy for autoimmunity or other inflammatory conditions.

## Supporting Information

Figure S1 **Comparison of inhibitory receptors expressed by enTregs and iTregs.** (A) Representative histograms generated using flow cytometric analysis of surface receptors expressed by iTregs (black line) isolated from the gastric lymph nodes TxA23 mice 1 week after treated and enTregs (gray filled line) from age matched untreated TxA23 mice. Data represents an aggregation of 2-4 individual experiments.
(TIF)

Figure S2 **iTregs upregulated surface receptors after transfer into TxA23 mice.** (A) Representative histograms generated using flow cytometric analysis of surface receptors on iTregs prior to transfer (gray filled line) and iTregs 1 week after transfer into TxA23 mice (black line). Data represents 2 independent experiments.
(TIF)

Figure S3 **Comparison of cytokines and transcription factors expressed by enTregs and iTregs.** mRNA was isolated from enTregs and iTregs that were transferred into TxA23 mice, analyzed by qRT-PCR and normalized to mRNA isolated from CD4^+^Foxp3^-^ T cells from BALB/c mice. (A) Comparison of expression levels of *Il10*, *Gata3*, and *Blimp1*. Data shown as mean ± SEM from 4-6 independent experiments and analyzed using a Mann–Whitney *U* test. *p<0.05, NS = not significant.
(TIF)
