# Supplementary figures and images for: In Vitro Induced Regulatory T Cells Are Unique from Endogenous Regulatory T Cells and Effective at Suppressing Late Stages of Ongoing Autoimmunity
Source: PLoS One. 2014 Aug 13;9(8):e104698. doi: 10.1371/journal.pone.0104698 (PMC4131893; doi:10.1371/journal.pone.0104698)

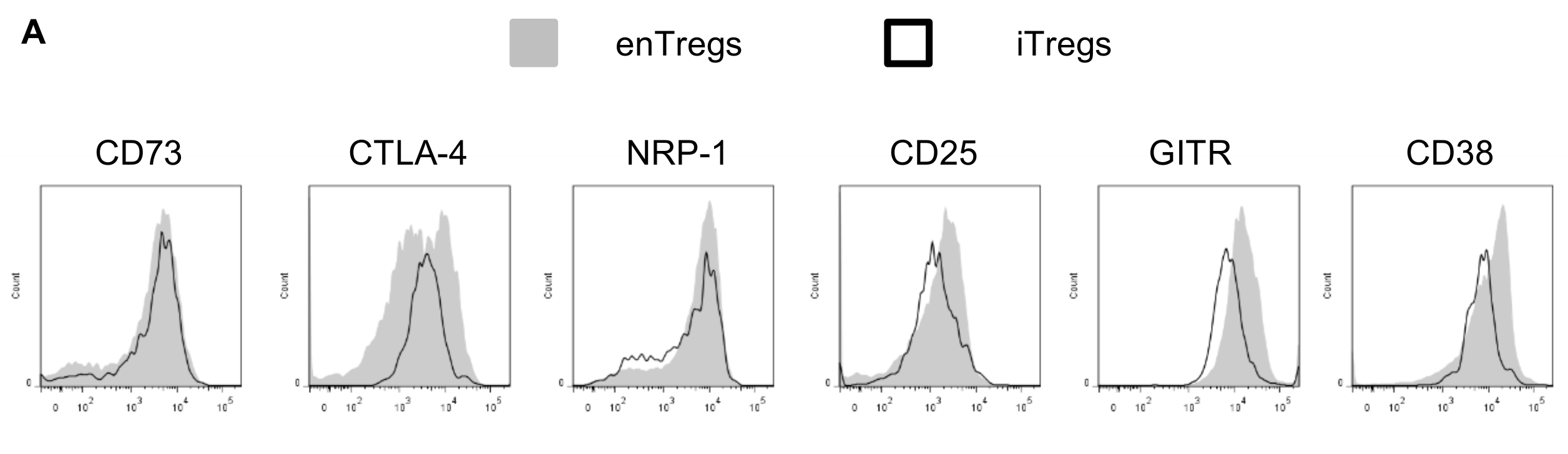

Supplement: Figure S1 — Comparison of inhibitory receptors expressed by enTregs and iTregs. (A) Representative histograms generated using flow cytometric analysis of surface receptors expressed by iTregs (black line) isolated from the gastric lymph nodes TxA23 mice 1 week after treated and enTregs (gray filled line) from age matched untreated TxA23 mice. Data represents an aggregation of 2-4 individual experiments. (TIF) [file pone.0104698.s001.tif]

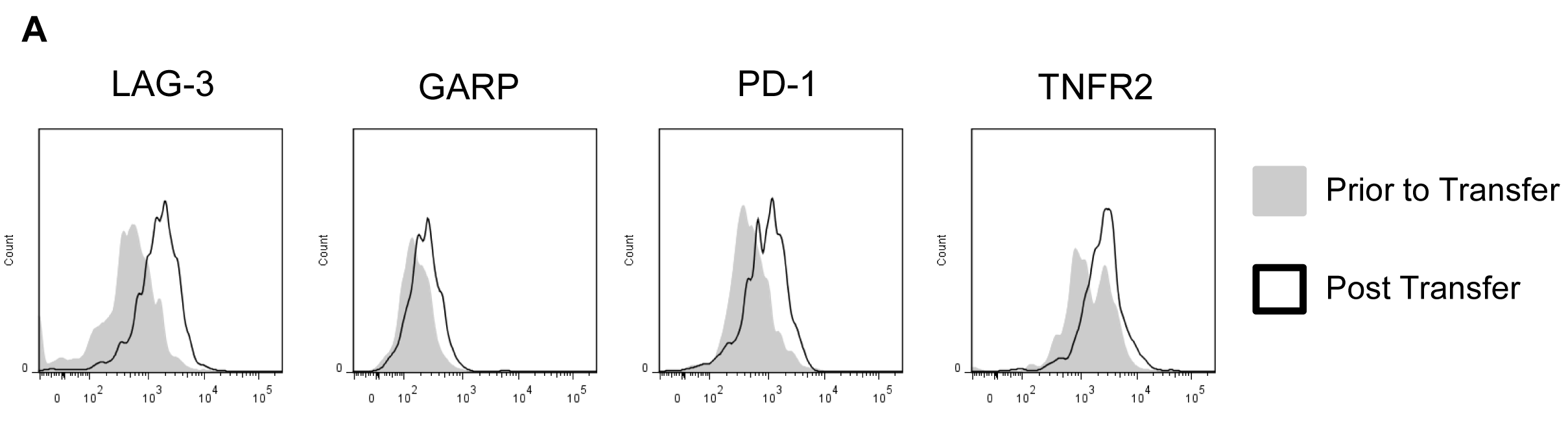

Supplement: Figure S2 — iTregs upregulated surface receptors after transfer into TxA23 mice. (A) Representative histograms generated using flow cytometric analysis of surface receptors on iTregs prior to transfer (gray filled line) and iTregs 1 week after transfer into TxA23 mice (black line). Data represents 2 independent experiments. (TIF) [file pone.0104698.s002.tif]

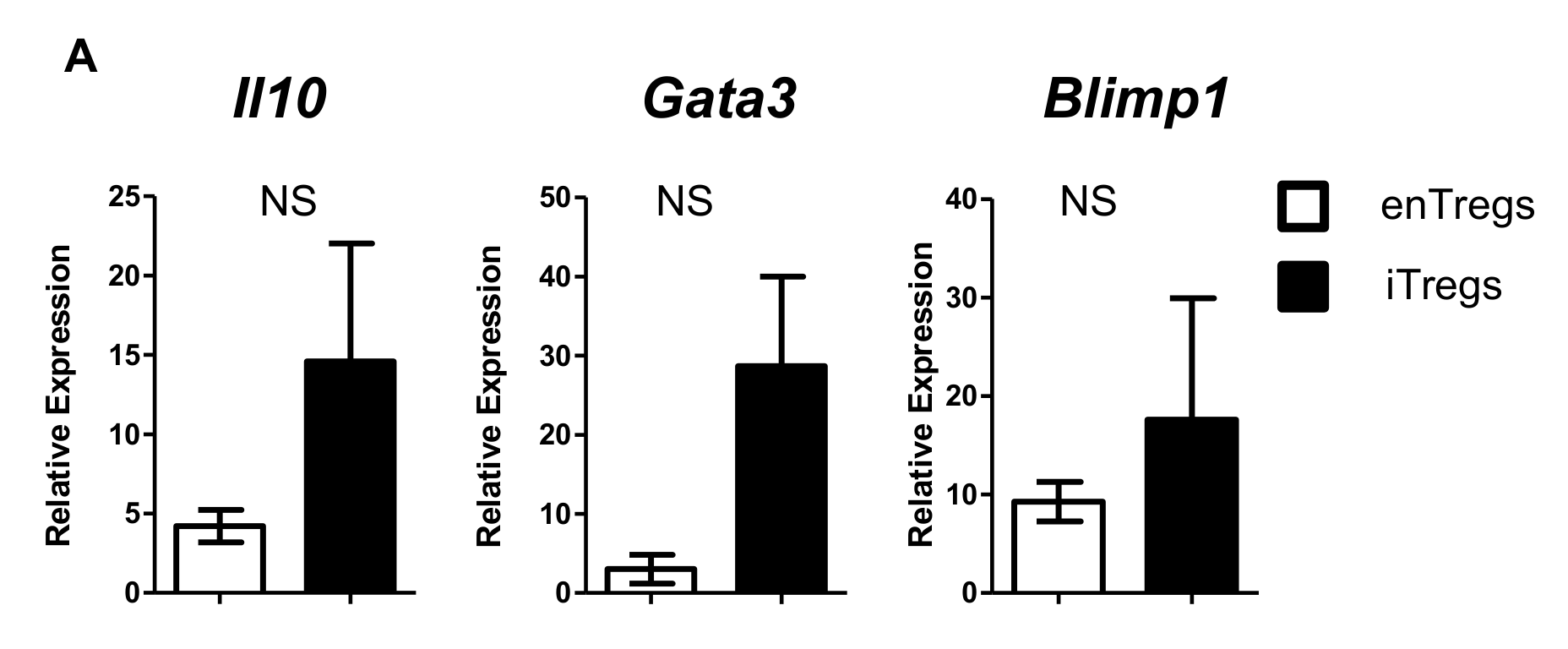

Supplement: Figure S3 — Comparison of cytokines and transcription factors expressed by enTregs and iTregs. mRNA was isolated from enTregs and iTregs that were transferred into TxA23 mice, analyzed by qRT-PCR and normalized to mRNA isolated from CD4+Foxp3- T cells from BALB/c mice. (A) Comparison of expression levels of Il10, Gata3, and Blimp1. Data shown as mean ± SEM from 4-6 independent experiments and analyzed using a Mann–Whitney U test. *p<0.05, NS = not significant. (TIF) [file pone.0104698.s003.tif]
